# The Medical Graduate and His Needs

**Published:** 1884-11

**Authors:** 


**The Medical Graduate and his Needs.** By GEORGE C. WELLNER, M. D. Detroit, Mich.: George S. Davis. 1884.

This little volume contains deductions of a careful observer from the practical experience of the young physician. It is intended to unravel certain difficulties in professional life and to smooth the pathway of obstructions. It contains many valuable hints, which we especially commend to the young practitioner. The work has a purpose, an object, which we are sure will be fully appreciated by those who are interested in this kind of medical literature.

